# Dyspnoea at rest predictor of increased in-hospital mortality in metastatic cancer patients undergoing emergent surgery in United States

**DOI:** 10.3332/ecancer.2020.1112

**Published:** 2020-09-24

**Authors:** Elleana J Majdinasab, Yana Puckett

**Affiliations:** West Virginia University School of Medicine, WV 25301, USA

**Keywords:** dyspnoea, in-hospital mortality, metastatic cancer, emergent surgery, prognosis, advanced cancer

## Abstract

**Background:**

Dyspnoea is an extremely common finding in patients presenting with metastatic cancer and can be caused by cancer progression, treatment toxicity or pathology secondary to deteriorating overall health. In this study, we decided to analyse post-operative outcomes to understand if dyspnoea is a significant prognostic predictor of in-hospital mortality in patients with stage IV cancer who underwent emergent surgery in the United States.

**Methods:**

We performed a search of the 2014 National Surgical Quality Improvement Program database (NSQIP) for patients with a diagnosis of malignancy (ICD-9 Codes 145.00–200.00). Cases were divided into two groups: metastatic cancer and non-metastatic cancer. Demographical data including preoperative, intraoperative and postoperative factors, as well as data regarding complications and comorbidities were compared between these two groups. Independent t-testing was used to compare continuous variables. Chi-square testing was used to compare categorical variables. Multiple logistic regression was used to assess for predictors of mortality in metastatic cancer. Mortality was adjusted for demographics, comorbid conditions and perioperative factors.

**Results:**

Referring to the NSQIP database, a total of 80,275 cancer patients were analysed, 11.8% (9,423) of whom had metastatic cancer. Dyspnoea at rest/moderate exertion (OR 5.7/2.4; 95% CI 2.7/1.6–11.9/3.7; p < 0.0001) were found to be the biggest predictors of in-hospital mortality in stage IV cancer patients who underwent emergent surgery.

**Conclusion:**

Dyspnoea at rest and with moderate exertion may be used as predictors of in-hospital mortality for metastatic cancer patients undergoing emergent surgery.

## Introduction

It can be extremely devastating, isolating and overwhelming for a patient to be diagnosed with cancer. This sentiment rings even louder for patients with metastatic cancer [1]. Metastatic cancer is the process by which tumour cells successfully disseminate from their primary site, enter the systemic circulation, and establish themselves in new locations [2].

Dyspnoea is a common symptom experienced by patients with metastatic cancer. Dyspnoea is the subjective and uncomfortable awareness of one’s ability to breathe [3]. It can be precipitated by pathology within the respiratory, musculoskeletal, cardiovascular, haematological or physiological system, referring to potentially any dysfunction in the body’s regulatory homeostatic mechanisms [4]. The American Thoracic Society defined it to be ‘a subjective experience of breathing discomfort that consists of qualitatively distinct sensations that differ in intensity.’ This definition, while dated, is made relevant with their addition of three evidence-based caveats which include the acknowledgement that (1) many mechanisms and pathways influence work of breathing, chest tightness, and air hunger, (2) individual such sensations usually do not occur in isolation and (3) the sensations of dyspnoea vary in terms of emotional and behavioural significance to the patient [5].

Prior studies have at length explored the possibility of dyspnoea’s prognostic value in patients with metastatic cancer. It has been established by the hallmark National Hospice study that 70.2% of terminally ill cancer patients experienced breathlessness during their last 6 weeks of life [6]. Muer *et al* [7] expanded on this by confirming that dyspnoea was more common in patients with non-small cell lung cancer (NSCLC), and went on to make the claim that dyspnoea prevalence increased as patients approached death; at diagnosis, only 60% of patients reported dyspnoea compared to a much higher 90% prior to death. Another, more conservative study due to the lack (only 4%) of lung cancer participants showed that dyspnoea was present in 49.1% of patients seen at a regional cancer centre. Dyspnoea in cancer patients has a multitude of causes. The same study classified causes of dyspnoea into four categories: direct tumour effects, indirect tumour effects, treatment related causes, and problems unrelated to the cancer [8].

With this knowledge, we decided to evaluate the prognostic role of dyspnoea in the context of in-hospital mortality for patients with metastatic cancer. The objective of this study is to determine if the presence of dyspnoea in metastatic cancer patients serves any value as a predictor for in-hospital mortality in those who underwent emergent surgery, wherein emergent surgery is defined to be any surgical intervention that occurred that was not originally intended to be a component of the patient’s treatment plan.

## Materials and methods

We performed a search of the 2014 National Surgical Quality Improvement Program database, a collection of reliable data from over 500 participating American hospitals that includes statistics on preoperative risk factors and postoperative complications, demographics, comorbidities and 30-day postoperative patient outcomes. The clinical data is standardised and validated by trained and certified surgical clinical reviewers specific to each hospital, using universal and standardised data parameters [9].

Patients with diagnoses of malignancy (ICD-9 145.00 to 200.00) were gathered and assembled into two groups depending primarily on whether they had metastatic or non-metastatic cancer. Demographical data, perioperative factors, and data on complications/comorbidities were compared between the two groups. Confounding variables that were adjusted for include age, gender, race, comorbid conditions and perioperative factors. Independent *t*-test was used to compare continuous variables and Chi-square test was used to compare categorical variables, specifically in-hospital mortality, unplanned return to the ER, organ/space surgical site infection (SSI), deep incisional SSI, superficial SSI, pneumonia, bleeding transfusions, myocardial infarction and sepsis.

All missing data was excluded in the statistical analysis. A homogeneity test was performed utilising SPSS statistical software packaging and data was confirmed to be homogeneous. Multiple logistic regression was used to assess for predictors of mortality in patients with metastatic cancer, which included unplanned return to the emergency room, male gender, dyspnoea at rest and at exertion, white blood cell count (WBC) and elevated alkaline phosphatase, along with diabetes mellitus type 2 (DM), smoking status, chronic obstructive pulmonary disease (COPD), ascites, congestive heart failure (CHF), hypertension (HTN), dialysis, wound infection, steroid use, weight loss, bleeding disorder, transfusion requirement, sodium, blood urea nitrogen (BUN), creatinine, albumin, bilirubin, haematocrit, platelets, partial thromboplastin time (PTT), time in operating room and emergency surgery. A *p* value of <0.05 was deemed to be statistically significant.

## Results

A total of 80,275 patients were analysed. Of those, 11.8% comprised the metastatic cancer group. Table 1 displays the surgical outcome data collected from the two patient groups.

Patients in the metastatic cancer group experienced four times increased incidence of mortality within the hospital setting relative to the non-metastatic cancer group in the same setting. It was found that 55.6% of patients in the metastatic group experienced unplanned returns to the operating room, compared to only 3.7% of the non-metastatic group, a 15-fold difference (*p* < 0.0001). Bleeding transfusions and sepsis occurred in 18.2% and 4.3% of the metastatic group, respectively, compared to 7.0% and 1.7% of the non-metastatic group, respectively (*p* < 0.0001). Other complications such as organ/space SSI and pneumonia also occurred at higher rates, 0.5% and 0.4% respectively, within the metastatic group, while only 0.1% of the non-metastatic group experienced such events (*p* < 0.0001).

After adjusting for confounding variables, such as age, gender, comorbidities and functional status unplanned return to the operating room (OR 2.7; 95% CI 1.7–4.3; *p* < 0.0001), male gender (OR 1.5; 95% CI 1.1–2.1; *p* = 0.009), dyspnoea at rest/moderate exertion (OR 5.7/2.4; 95% CI 2.7/1.6–11.9/3.7; *p* < 0.0001), elevated alkaline phosphatase levels (OR 1.001; 95% CI 1-1.003; *p* = 0.02), WBC (OR 1.025; 95% CI 1.013–1.037; *p* < 0.0001) and elevated INR levels (OR 1.006; 95% CI 1.001–1.01; *p* = 0.025) were associated with increased odds of mortality for metastatic cancer patients admitted to the hospital (Table 2). Of all the patients admitted with metastatic cancer, 18.5% underwent emergent surgery.

## Discussion

The primary focus of this study was to evaluate the surgical outcomes of metastatic cancer patients in the United States. We found that patients with metastatic disease who died while in the hospital status post-surgery were 5.6 times more likely to have dyspnoea at rest and 2.4 times more likely to have dyspnoea on moderate exertion. Although shortness of breath is not uncommon and occurs in up to 78.6% of advanced cancer patients, this finding makes sense, given that dyspnoea increases in severity and frequency as cancer progresses and the patient nears death [10]. Our data supports prior studies that detailed that shortness of breath is a symptom commonly experienced in patients diagnosed with metastatic cancer.

More importantly, our findings are consistent with prior literature regarding the prognostic role of dyspnoea in advanced cancer patients. Both dyspnoea on rest and moderate exertion were associated with higher incidence of mortality while in the hospital (Table 2). One notable 2016 study focused on dyspnoea upon initial presentation of NSCLC patients and found significant prognostic value in the metric. They discovered that median survival in NSCLC patients with dyspnoea was significantly shorter (7.9 months) than those without (15.3) [11]. This finding is noteworthy in that clinicians, both in the ER and oncology clinic, may know to place more weight on shortness of breath as either a chief complaint or associated symptom in the history of present illness. It may serve to alert physicians to spend more time on and pay more focused attention to these patients than previously thought, potentially second-guessing premature discharge, with the goal of increasing chances of prolonged survival.

Another study, this time observing hospice admissions in the United Kingdom, performed by Heyse-Moore *et al* [12] similarly demonstrated the fact that dyspnoea is a predictor of poor prognosis, with a prevalence of dyspnoea in 55.5% of patients before increasing to 78.6% in patients who ultimately passed. These results were similar to that of Muer *et al* [7] regarding the increase in prevalence of dyspnoea as patients neared death, which our findings remain with.

Our results also correlate closely with those of a study performed by Edmonds *et al* [13] that demonstrated that advanced cancer patients with dyspnoea were more likely to die in the hospital than at a nursing home or hospice care. In our study, the mortality rate in patients with metastatic disease residing within the hospital setting was four times more frequent than that of patients with non-metastatic cancer. In addition, our findings showed that metastatic cancer patients were 15 times more likely to undergo emergent surgery compared to non-metastatic patients. This should not be surprising, given the multitude of complications that can arise secondary to comorbidities, cancer, and its aggressive treatment. As metastatic cancer is disseminated, the damage it causes to the human body is relatively severe compared to localised, non-metastatic cancer. As such, progression of the disease and aggressive treatment increase the likelihood for complications to arise that must be treated with emergent surgery. It is also probable that the terminally ill metastatic cancer patients undergoing emergent surgery are those who opt for extraordinary life-preserving measures.

While we did not analyse imaging or pulmonary function testing in our current study, Dudgeon *et al* [4] reported 91% of advanced cancer patients involved demonstrated abnormal chest radiography, and 93% had abnormal pulmonary function tests. We also did not group the patients involved in our study by division of malignancy, but it is of note that Muers *et al* [7] found that shortness breath was more common in patients with NSCLC.

Our study further reveals that patients with metastatic cancer develop complications like pneumonia, organ/space SSI, and sepsis more frequently than do patients with non-metastatic cancer. Cancer and its treatment leave patients vulnerable to such complications [14]. Our study showed that the incidence of pneumonia in metastatic cancer patients after surgery was four times that of non-metastatic patients (Table 1). The development of nosocomial pneumonia in vulnerable cancer patients may be a potent contributor to the dyspnoea that is experienced. Therefore, clinicians may want to acquire heightened awareness of respiratory symptoms experienced by metastatic cancer patient’s status post surgery. Efforts to mitigate nosocomial pneumonia via prophylaxis may also be considered to prevent the onset of dyspnoea, given that it is a poor prognostic predictor.

One limitation to this study was that we did not distinguish between the myriad sensory perceptions and presentations of dyspnoea, such as tightness, pressure or heaviness, for example. Another limitation is the mere fact that dyspnoea is a generalised, broad and subjective symptom. Apart from pulse oximetry, the symptom is difficult to objectively measure. Each patient suffers from and therefore describes their experience with shortness of breath uniquely. Furthermore, our observations were broad in the context of cancer in that we did not group our study participants by type of malignancy. It is also challenging to pinpoint the exact cause of dyspnoea if there are no overt findings on imaging or laboratory values; in such cases, one might consider psychogenic causes in the differential diagnoses. The data does not provide information on the type of surgery performed, available surgical expertise which could certainly influence the outcomes of the patients. This is a limitation of our study and a limitation of using a large database. Finally, we did not collect or incorporate values of pulmonary function into our study.

## Conclusion

The presence of dyspnoea among patients with metastatic cancer appears to be a reliable prognostic metric for in-hospital mortality. Clinicians may utilise this information to provide swift and targeted care to dyspnoeic cancer patients to improve survival outcomes and life expectancy. Specifically, physicians may begin to interpret dyspnoea as a type of alarm that prompts them to act quickly with appropriate interventions to decrease their patients’ chances of in-hospital mortality. Alternatively, physicians may appreciate having increased intuition for their patients’ prognoses such that they can modify treatment plans to instead reflect palliative care in lieu of aggressive treatment.

## Funding

The authors did not receive funding for this study.

## Conflicts of interest

The authors declare that they have no conflicts of interest.

## Figures and Tables

**Table 1.** Comparison of surgical outcomes between patients with metastatic cancer and non-metastatic cancer (*n* = 80,275).

	Metastatic (*n* = 9,423)	Non-metastatic (*n* = 70,852)	*p*-value
In hospital mortality	2.1% (195)	0.5% (371)	<0.0001
Unplanned return to the operating room	55.6% (524)	3.7% (2,621)	<0.0001
Organ/Space SSI	0.5% (47)	0.1% (93)	<0.0001
Deep incisional SSI	0.1% (10)	0.02% (18)	0.001
Superficial SSI	0.1% (13)	0.04% (35)	0.003
Pneumonia	0.4% (34)	0.1% (76)	<0.0001
Bleeding transfusions	18.2% (1,714)	7.0% (4,963)	<0.0001
Myocardial infarction	0.7% (65)	0.5% (336)	0.005
Sepsis	4.3% (404)	1.7% (1,224)	<0.0001

**Table 2.** Multiple logistic regression with predictors of in hospital mortality in patients with metastatic cancer.

	Beta coefficient	Odds	95% CI.		*p*-value
			Lower	Upper	
Unplanned return to operating room	1.004	2.729	1.749	4.256	<0.0001
Male gender	0.414	1.513	1.11	2.063	0.009
Insulin dependent DM2	−0.901	0.406	0.172	0.962	0.04
Non-insulin dependent DM2	0.035	1.035	0.627	1.71	0.892
Smoker	−0.107	0.899	0.626	1.291	0.564
Dyspnoea (at rest)	1.734	5.662	2.7	11.87	<0.0001
Dyspnoea (moderate exertion)	0.88	2.411	1.591	3.653	<0.0001
COPD	−0.701	0.496	0.305	0.807	0.005
Ascites	−1.021	0.36	0.225	0.576	<0.0001
CHF	−0.121	0.886	0.337	2.332	0.807
HTN	−0.296	0.744	0.544	1.018	0.064
CHF	1.259	3.521	0.688	18.006	0.131
On dialysis	−2.073	0.126	0.047	0.334	<0.0001
Wound infection	−0.493	0.611	0.314	1.189	0.147
On steroids	−0.128	0.88	0.526	1.474	0.627
Weight loss	−0.147	0.863	0.571	1.305	0.486
Bleeding disorder	−0.537	0.584	0.354	0.965	0.036
Requirement for transfusion	−0.305	0.737	0.423	1.284	0.282
Na level	−0.004	0.996	0.99	1.003	0.28
BUN level	0.007	1.007	0.999	1.015	0.103
Creatinine level	0.01	1.01	0.989	1.033	0.353
Albumin level	−0.001	0.999	0.992	1.006	0.773
Bilirubin level	0	1	0.992	1.008	0.987
Alkaline phosphatase	0.001	1.001	1	1.003	0.019
WBC	0.025	1.025	1.013	1.037	<0.0001
Haematocrit	−0.026	0.975	0.964	0.985	<0.0001
Platelets	0	1	0.999	1.002	0.515
PTT	−0.001	0.999	0.996	1.002	0.37
INR	0.006	1.006	1.001	1.01	0.025
PTT	−0.151	0.86	0	1.87E+179	0.999
Time in operating room	0.001	1.001	1	1.002	0.168
Emergency surgery	−0.726	0.484	0.308	0.76	0.002
